# Associations between Adverse Childhood Experiences and Sexual Risk among Postpartum Women

**DOI:** 10.3390/ijerph18073848

**Published:** 2021-04-06

**Authors:** Jordan L. Thomas, Jessica B. Lewis, Jeannette R. Ickovics, Shayna D. Cunningham

**Affiliations:** 1 Department of Psychology, University of California Los Angeles (UCLA), Los Angeles, CA 90095, USA; thomasjl@ucla.edu; 2 Department of Chronic Disease Epidemiology, Yale School of Public Health, New Haven, CT 06510, USA; jessica.lewis@yale.edu; 3 Yale-NUS College, Singapore 138527, Singapore; jeannette.ickovics@yale-nus.edu.sg; 4 Department of Social and Behavioral Sciences, Yale School of Public Health, New Haven, CT 06510, USA; 5 Department of Public Health Sciences, University of Connecticut School of Medicine, Farmington, CT 06032, USA

**Keywords:** adverse childhood experiences, postpartum women, sexual risk, contraceptive behavior, condoms, rapid repeat pregnancy, sexually transmitted infections

## Abstract

Epidemiological evidence suggests that exposure to adverse childhood experiences (ACEs) is associated with sexual risk, especially during adolescence, and with maternal and child health outcomes for women of reproductive age. However, no work has examined how ACE exposure relates to sexual risk for women during the postpartum period. In a convenience sample of 460 postpartum women, we used linear and logistic regression to investigate associations between ACE exposure (measured using the Adverse Childhood Experiences Scale) and five sexual risk outcomes of importance to maternal health: contraceptive use, efficacy of contraceptive method elected, condom use, rapid repeat pregnancy, and incidence of sexually transmitted infections (STIs). On average, women in the sample were 25.55 years of age (standard deviation = 5.56); most identified as Black (60.4%), White (18%), or Latina (14.8%). Approximately 40% were exposed to adversity prior to age 18, with the modal number of experiences among those exposed as 1. Women exposed to ACEs were significantly less likely to use contraception; more likely to elect less-efficacious contraceptive methods; and used condoms less frequently (*p* = 0.041 to 0.008). ACE exposure was not associated with rapid repeat pregnancy or STI acquisition, *p >* 0.10. Screening for ACEs during pregnancy may be informative to target interventions to reduce risky sexual behavior during the postpartum period.

## 1. Introduction

Adverse childhood experiences (ACEs)—inclusive of exposures ranging from household dysfunction (e.g., household member with substance abuse, parental absence due to incarceration) to abuse (psychological, physical, sexual)—are prevalent, deleterious, and important social determinants of subsequent health. While much research has identified the health consequences of ACEs more broadly [1,2], a burgeoning body of work has focused specifically on documenting the effects of these exposures among perinatal populations, and increasing evidence suggests adverse impacts for maternal and child health.

Women exposed to ACEs are over-represented among those who have adverse birth outcomes, including miscarriage [3,4], shortened gestational period and low birthweight [5,6]. They also are at increased risk for perinatal depression and anxiety [7,8] as well as other chronic health conditions [9]. The impact of maternal ACEs extends beyond biology to behavior, including substance use during pregnancy [10,11,12] and breastfeeding [13], and effects on offspring have been documented, as well. The children of women exposed to ACEs are at-risk for neurodevelopmental challenges, including higher levels of externalizing and internalizing problems [14]. Moreover, emerging work suggests that maternal exposure to ACEs may have epigenetic effects [15,16], through biological (e.g., hypothalamic-pituitary-adrenal axis alteration during pregnancy [17]) and/or environmental (e.g., maternal stress during pregnancy [9]) pathways, which poses increased risk for fetal development while in utero. Increasing empirical [18] and clinical [19] recognition of the risks of intergenerational transmission of maternal adversity suggests that further study of ACEs is needed to appropriately intervene to improve maternal and child health.

Research among adolescents and young women demonstrates associations between early adversity and markers of adolescent sexual risk, including earlier age of first sex [20,21], election of a greater number of sexual partners [20,21,22], and incidence of sexually transmitted diseases [20,22]. Additionally, ACEs have been linked with unintended [23,24,25] and adolescent [26] pregnancy, as well as with reproductive intentions more broadly [25,26]. Less is known about the association between exposure to adversity and sexual risk among the full range of women of reproductive age. No research to date has assessed the impact of early adversity on sexual risk behaviors among postpartum women specifically, for whom sexual risk poses unique concerns.

Postpartum contraceptive use is an important strategy for preventing unintended pregnancy and optimizing birth spacing, yet nearly 40% of the 61 million women of reproductive age in the United States (US) report not using any contraceptive method, and only one-quarter (26.2%) report using highly efficacious methods [27]. High-efficacy contraceptives—including long-lasting reversible contraception (LARC)—have been shown to optimize interpregnancy intervals [28,29], whereas sole reliance on low-efficacy contraceptives (e.g., barrier methods) increases risk for shorter birth spacing and subsequent pregnancy [29]. Rapid repeat pregnancy (becoming pregnant again within 18 months of a delivery) increases risk for both pregnant women (e.g., anemia, uterine rupture) and their neonates (preterm birth, low birthweight) [30,31,32]. The Healthy People 2030 initiative recently named both optimization of birth spacing and prioritization of effective methods of contraception for reproductive-aged women as two objectives for the next decade—reflecting a national priority for sexual risk reduction among this population [33].

As a barrier method, condoms—whether used on their own or in conjunction with other contraceptives—have the added benefit of protecting against sexually transmitted infections (STIs). STIs are critical to prevent among postpartum women because of the health risks they pose (e.g., cervical cancer, infertility, increased HIV risk) to women and harm they may cause to a subsequent pregnancy (e.g., preterm birth, ectopic pregnancy, congenital infection) [34]. Women are easily accessible for STI prevention interventions during the perinatal period [35], a time when they are typically more connected to the health system than during any other healthy period of their lives.

We present the first investigation to assess associations between ACE exposure and postpartum sexual risk markers (contraceptive use, efficacy of contraceptive method elected, condom use frequency, rapid repeat pregnancy, and STI incidence) among a diverse cohort of women of childbearing age during the year following delivery. We predicted that adversity-exposed women would demonstrate riskier sexual behavior—including use of low-efficacy contraceptives and less consistent condom use—as well as be at increased risk for rapid repeat pregnancy and STI acquisition one-year postpartum. Elucidating predictors of sexual risk behaviors and their clinical consequences will help better identify women who may benefit from targeted intervention. Moreover, pregnant and postpartum women are uniquely positioned to be screened for ACEs, given their frequent contact with the healthcare system for prenatal and pediatric care, and recent research suggests that ACE screening in the context of prenatal care may be feasible for both patients and providers [36,37]. Should ACE exposure be related to markers of sexual risk among postpartum women, appropriate risk reduction interventions may be similarly disseminated through these mechanisms.

## 2. Materials and Methods

### 2.1. Study Population

Data for these analyses were obtained from a prospective longitudinal cohort study of Expect With Me, a group prenatal care model aimed at improving maternal health and birth outcomes [38]. Between 2014 and 2017, a convenience sample of women attending three prenatal care clinics in Detroit, MI and Nashville, TN in the US were referred by a health care provider for participation in the study. Eligibility criteria included entering care before 24 weeks gestation at a participating prenatal practice; ability to speak English or Spanish; and willingness to participate in the study. Staff at each clinical site explained the study to eligible participants, answered questions, and obtained informed consent. Participating women completed online surveys during their second trimester of pregnancy, third trimester of pregnancy, at the birth of their child, and at six and twelve months postpartum, and were paid $20 for each interview. The Institutional Review Boards at Yale, Vanderbilt, and Wayne State Universities approved all study procedures.

Analyses for this paper used data collected during the second trimester of pregnancy, as well as at six- and twelve-months postpartum. The cohort is limited to study participants with ACEs data, resulting in an analytic sample of 460 women. Compared to women included in this analytic sample, those excluded were more likely to be younger, *p* < 0.05. There were no other significant sociodemographic differences.

### 2.2. Measures

Exposure to ACEs was assessed using the Adverse Childhood Experiences Scale [1]. Women reported exposure to a series of negative life circumstances prior to age 18, including experiences of physical, psychological, or sexual abuse; emotional and physical neglect; witnessing domestic violence; separation from a parental figure through divorce, abandonment, or imprisonment; or living with a household member with a mental health or substance use problem. Given that adversities often co-occur [39,40], we dichotomized the exposure variable to represent any exposure to adversity versus none.

Three sexual risk behaviors were assessed using a sexual risk questionnaire designed by the authors and used in studies of women during the perinatal period [41]. To capture contraceptive use, participants were asked whether they had used particular contraceptive methods in the past six months for birth control or protection against sexually transmitted infections, including condoms, birth control pill, Depo-Provera^®^ shot, other hormonal birth control (patch, ring, implant), intrauterine device (IUD), or other (e.g., tubes tied, withdrawal). We dichotomized contraceptive use to indicate those who used any method and those who used none. We also categorized contraceptive methods as “low-efficacy (for birth control) or no contraceptives” (e.g., barrier and natural methods) or “high/moderate-efficacy contraceptives,” (e.g., hormonal methods), using Centers for Disease Control and Prevention classifications [42]. Those who reported using multiple types of contraception were categorized by their most effective method [43]. For condom use frequency, women who reported using condoms as a method were asked what percent of the time they used condoms when having sex, ranging from 0% (never) to 100% (always), as in previous research [41].

Two biological outcomes were also assessed: rapid repeat pregnancy (within 12 months of giving birth [44]) and postpartum STI incidence. Women reported whether they had become pregnant since their index pregnancy. Additionally, they were asked if they were diagnosed with any of the following STIs since their delivery: chlamydia, gonorrhea, genital warts or human papilloma virus, herpes, syphilis, or trichomonas.

Covariates consisted of sociodemographics; women reported their date of birth (from which age in years was calculated), race and ethnicity, whether they had no/public or private health insurance, and whether they were currently in a romantic or sexual relationship.

### 2.3. Data Analyses

Analyses were conducted using SPSS Version 27 (IBM Corporation, Armonk, NY, USA) [45]. Means and frequencies were calculated to characterize the sample. Logistic regression analyses were used to test associations between ACE exposure and binary outcomes, including any contraceptive use, low-efficacy or no contraceptive use (versus high/moderate-efficacy), and incidence of repeat pregnancy and STIs. Linear regression analyses were used to assess associations between ACE exposure and frequency of condom use. Analyses controlled for known predictors of sexual risk, including age, race and ethnicity, private versus no/public health insurance (socioeconomic indicator), and relationship status [46], as well as for study site.

## 3. Results

### 3.1. Participant Characteristics

As seen in Table 1, the sample was racially and ethnically diverse, with many women identifying as Black (60.4%) or Latina (14.8%). Overall, women were around 25 years of age, and most (70.7%) had either public or no health insurance. Approximately 40% of the sample had experienced one or more adverse childhood experience, with a full range of 0–10 exposures reported; among those exposed, the modal number of experiences endorsed was 1. The most commonly reported ACEs were separation from a biological parent or primary caretaker and household member with substance use, each of which was endorsed by 15% of the sample. Nearly 70% of women reported using some form of contraception; 50.7% were using high- or moderate-efficacy methods, and methods were not mutually exclusive. Approximately eleven percent (10.7%) were pregnant again by the one-year postpartum assessment. Self-reported STI incidence was low (2.4%).

### 3.2. Associations between ACE Exposure and Sexual Risk

Women exposed to ACEs were less likely to use any contraception (adjusted odds ratio [AOR] = 0.57; 95% confidence interval [CI] = 0.38, 0.86) and more likely to rely on contraceptive methods that had low-efficacy for birth control (including using no contraception) versus high/moderate efficacy (AOR = 1.50, 95% CI = 1.02, 2.21) (Table 2). Among women using condoms, those with a history of ACEs demonstrated less consistent condom use (β = −15.81; standard error = 6.85) relative to those not adversity-exposed. ACE exposure was not significantly associated with rapid repeat pregnancy (AOR = 1.41, 95% CI = 0.75, 2.63) or STI incidence (AOR = 1.43 95% CI = 0.42, 4.84).

We considered that women currently in a romantic or sexual relationship or those who reported recent sexual activity may be driving these effects. We performed a sensitivity analysis and saw a similar pattern of findings when we restricted analyses to only those women who reported currently being in a romantic or sexual relationship (*n* = 359) and those women who reported one or more male sexual partners (*n* = 402). Restricting the sample did not change the direction or significance of results.

## 4. Discussion

This study found that a diverse cohort of postpartum women who were exposed to adverse childhood experiences were more likely to engage in risky sexual behavior than those not adversity-exposed. The proportion of women exposed to ACEs in this study (40%) was comparable to estimates researchers have found in some perinatal samples (e.g., 46% [37]), but not as high as in others (e.g., 69% [36]), which may reflect demographic and regional differences in study populations. We observed significant associations between adversity exposure and contraceptive behavior, including use of any contraceptive, the efficacy of the method of contraception selected, and the frequency of condom use among those using condoms. However, we did not see an effect of ACEs on rapid repeat pregnancy or on STI acquisition. These biological outcomes require contraceptive failure during a period of ovulation and having sex with an infected partner, respectively, and thus are more distal endpoints and more difficult associations to detect. Further, incidence of both of these clinical outcomes was low in this sample (10.7% and 2.4%, respectively), which may have muted our ability to detect significant differences by ACE exposure.

The perinatal period represents a window of opportunity for prevention [47]. Women are more connected to the healthcare system during the perinatal period than at any other time during the reproductive lifecycle, marking this window as an ideal time during which to deliver messages about and interventions promoting contraceptive use. Contraceptive use postpartum is important to prevent unintended pregnancy, optimize birth spacing if planning a subsequent pregnancy, and to prevent STIs. Leading domestic and international health organizations recommend that counseling on postpartum contraception be initiated during prenatal care [48] and that postpartum women be offered both contraceptive counseling and provision before discharge following childbirth [49]. Although not all contraceptive methods are recommended in the early postpartum period, the American College of Obstetricians and Gynecologists (ACOG) advises immediate postpartum LARC insertion—completed before hospital discharge—and notes few contraindications [50]. Our study findings suggest that women exposed to ACEs may particularly benefit from providers incorporating immediate postpartum LARC into their clinical practice, as well as from additional education on the efficacy of condoms as an STI prevention strategy when engaging in postpartum sexual activity.

Our study contributes to the growing evidence base linking ACE exposure with adverse health outcomes among perinatal populations. A growing number of researchers have applied a life-course perspective to the study of maternal and child health [6,51], due, in part, to increased recognition of the intergenerational transmission of maternal adversity and its sequelae. Greater understanding of the risks that ACE exposure poses both to adversity-exposed women and their infants is critical for appropriate assessment and intervention efforts to improve maternal and child health. In this study, we establish associations between exposure to ACEs and poor contraceptive behavior during the initial year postpartum. These results provide further evidence for advocating for increased ACE screening and longer-term follow-up of perinatal women, in order to better identify women at highest risk for risky sexual behavior and those who may need additional intervention or support.

Efforts to roll-out ACE screening in healthcare settings have begun to receive increasing attention in the US, particularly in California, where the *ACEs Aware Initiative* is spearheading screening and provider reimbursement efforts for pediatric and adult Medi-Cal patients [52]. We applaud policymakers for these efforts and, given our results and those of others elucidating the long-term impact of maternal childhood adversity on maternal and child health [3,4,5,6,7,8,9,10,11,12,13,14], call for similar consideration of screening for maternal ACEs among perinatal women across the US. Screening for ACEs and other psychosocial concerns have been shown to be feasible and acceptable to perinatal patients and providers [36,37], and administering these screenings in the context of perinatal care may hold unique clinical leverage in helping identify adversity-exposed women and direct them to targeted intervention.

This study has limitations. Adversity exposure was retrospectively reported, and while this methodology is typical within the ACEs literature, evidence suggests this approach captures different subsets of individuals than those who prospectively report it [53]. Further, our assessment window captured sexual risk outcomes reported by women through one year postpartum; while this timeframe has been used in previous research on sexual risk [44], it is possible that additional women may have become pregnant shortly thereafter, leading to an underreporting of rapid (within 18 months) repeat pregnancy. Additionally, available study data preclude our ability to investigate potential explanatory mechanisms linking ACE exposure and sexual risk (e.g., sexual education, sexual risk behavior prior to pregnancy). Data were also not available to assess degree of acculturation across ethnic or cultural groups. Future research should aim to include assessments of women’s attitudes toward sexuality, their exposure to sexual health education, and other pertinent variables.

This study also had several strengths. Most extant work linking ACEs and sexual risk has been conducted in adolescents [20,21,22,26]. We collected data from a large and diverse cohort of postpartum women who were not selected for adversity or trauma exposure. We focus both on clinical outcomes (e.g., rapid repeat pregnancy) and their behavioral precursors (e.g., contraceptive behavior), the latter of which may be particularly amenable to targeted and timely clinical intervention.

Prenatal care interventions that incorporate sexual risk reduction (e.g., group prenatal care models such as Expect With Me [38] or CenteringPregnancy Plus [44]) may potentially increase contraceptive uptake and consistent condom use among ACE-exposed women during the postpartum period. Other, more ACE-focused perinatal interventions—such as Perinatal Child–Parent Psychotherapy, which aims to target maternal experiences of trauma and adversity prior to delivery through psychotherapeutic techniques [54]—may similarly result in downstream effects on women’s sexual risk. Additional research is needed to evaluate the effect, if any, of extant interventions. Although we establish an association between adversity and sexual risk among postpartum women, a population for whom sexual risk poses acute concerns for both maternal and child health, future research should also examine these relationships among women at various stages of the reproductive lifecycle (e.g., during young adulthood, prior to pregnancy, among older adults). Using a longitudinal approach to investigate these associations among women of diverse ages may illuminate potential differences in how adversity exposure impacts sexual risk across the lifespan.

## 5. Conclusions

Exposure to adverse childhood experiences is associated with postpartum women’s sexual risk behavior—namely, poor contraceptive use—in the year following delivery. The perinatal period is an important window of opportunity during which to capitalize on identifying and addressing ACEs and their negative sequelae in the service of optimizing maternal and child health. Perinatal women should be screened for ACE exposure, and those identified should receive targeted interventions to offset potential sexual risk.

## Figures and Tables

**Table 1:** Sample Characteristics: Demographics, Adverse Childhood Experiences and Sexual Risk (*n* = 460).

**Sociodemographic and Relationship Characteristics**	***M* (*SD*) or % (*n*)**
Age at study enrollment (14–42)	25.55 (5.56)
Race and ethnicity	
Black, non-Hispanic	60.4 (278)
White, non-Hispanic	18.0 (83)
Latina	14.8 (68)
Other/refuse to answer	6.7 (31)
Study site	
Tennessee	45.2 (208)
Michigan	54.8 (252)
Insurance	
Public/None	70.7 (324)
Private	29.3 (134)
Currently in a romantic or sexual relationship	78.2 (359)
1 or more male sexual partners	88.9 (402)
**Adverse Childhood Experiences (ACEs) Exposures**	
ACE exposure—Any	40.7 (187)
Psychological abuse	13.9 (64)
Physical abuse	10.2 (47)
Sexual abuse	11.5 (53)
Emotional neglect	14.3(66)
Physical neglect	5.9 (27)
Divorce, abandonment	15.1 (69)
Witness domestic violence	8.5 (39)
Household member issue with using substances	15.0 (69)
Household member with mental illness	11.3 (52)
Household member incarcerated	7.6 (35)
**Sexual Risk Outcomes**	
Any contraceptive use, 12 months postpartum	69.1 (318)
Condoms	32.0 (147)
Birth control pill	12.8 (59)
Depo shot	15.9 (73)
Other hormonal birth control (e.g., patch, implant, ring)	9.6 (44)
IUD	12.0 (55)
Other (self-report; e.g., natural family planning, partner vasectomy)	6.3 (29)
Contraceptive efficacy	
High/moderate-efficacy contraceptives	50.7 (233)
Low-efficacy or no contraceptives	49.3 (227)
Condom use frequency (range 0–100)	67.69 (36.82)
Repeat pregnancy, up to 12 months postpartum	10.7 (49)
STI diagnosis	2.4 (11)

Note: IUD = intrauterine device; STI = sexually transmitted infection.

**Table 2:** Associations between ACEs exposure and sexual risk

	B (E) or AOR (95% CI)	*p*-Value
Any contraceptive use	0.57 (0.38, 0.86)	0.008
Low-efficacy contraceptive use	1.50 (1.02, 2.21)	0.041
Condom use	−15.81 (6.85)	0.023
Rapid repeat pregnancy	1.41 (0.75, 2.63)	0.282
STI incidence	1.43 (0.42, 4.84)	0.568

Note: B = unstandardized beta; E = standard error; AOR = adjusted odds ratio; CI = confidence interval; STI = sexually transmitted infection. Analyses adjusted for age, race and ethnicity, study location, relationship status at 12 months postpartum, and insurance coverage.

## Data Availability

Data are available to qualified researchers upon reasonable request of the authors.
